# Positive relationship between education level and risk perception and behavioral response: A machine learning approach

**DOI:** 10.1371/journal.pone.0321153

**Published:** 2025-04-03

**Authors:** Zhipeng Wei, Zhichun Zhang, Liping Guo, Wenjie Zhou, Kehu Yang

**Affiliations:** 1 Evidence-based Medicine Center, School of Basic Medical Sciences, Lanzhou University, Lanzhou, China; 2 Center for Evidence-based Social Science, School of Public Health, Lanzhou University, Lanzhou, China; 3 Innovation Laboratory of Evidence-based Social Science, Lanzhou University, Lanzhou, China; 4 School of Information Resource Management, Renmin University of China, Beijing, China; Rikkyo University: Rikkyo Daigaku, JAPAN

## Abstract

This paper aims to examine the influence mechanism of education level as a key situational factor in the relationship between risk perception and behavioral response, encompassing both behavioral intention and preparatory behavior. Utilizing non-parametric estimation techniques in machine learning, particularly the Random Forest and XGBoost algorithms, this study develops predictive models to analyze the impact of 27 influencing factors on behavioral responses following risk perception. The findings indicate that, while the model’s fit for preparatory behavior is 25.71% and its fit for behavioral intention is below 20%, the model effectively identifies key influencing factors. Further analysis employing SHAP values demonstrates that education level not only exerts a significant influence but also exhibits varying effects across different educational groups. Moreover, statistical testing corroborates the importance of education level in the relationship between risk perception and behavioral response, providing a robust scientific foundation for the development of risk management policies.

## 1. Introduction

Risk perception and behavioral response are vital elements of decision-making under uncertainty, especially in situations such as natural disasters, public health emergencies, and environmental threats. It’s crucial to understand the mechanisms that affect these processes to create effective risks management strategies. Among the numerous factors that influence risk perception and behavioral response, education level has been identified as a significant contextual variable. Nevertheless, the specific mechanisms by which education level impacts these outcomes are still not well understood, particularly in complex real-world situations where multiple variables interact in dynamic ways.

Risk perception has long been a central focus in disaster management and behavioral science. According to Siegrist and Arvai [1], research on risk perception can be broadly classified into three primary approaches: the characteristics of hazards, the characteristics of risk perceivers, and the application of heuristics in risk judgments.

These various perspectives contribute to a deeper understanding of how individuals assess disaster risks based on factors such as the controllability, predictability, and potential losses associated with hazards. Empirical studies in both natural and human-made disasters demonstrate that individuals’ perceptions of risk are shaped not only by the intrinsic characteristics of the disaster itself but also by emotional and cultural influences. For example, Mitsushita et al. [2] discovered that the cognitive frameworks for evaluating disaster risks differ between laypeople and experts, with the former exhibiting stronger emotional responses, such as dread and fear, towards certain hazards.

As demonstrated by Bodas et al. [3] in a cross-country study, individuals perceive risks associated with various disaster types (e.g., pandemics, extreme weather events, infrastructure failures) in diverse ways, and these perceptions, in turn, significantly influence their emergency response behaviors.

Risk perception is a complex, multidimensional construct that encompasses both cognitive evaluations of risk (e.g., likelihood of occurrence) and emotional responses (e.g., dread, fear, or uncertainty). As demonstrated by Bodas et al. [3] in a cross-country study, individuals perceive risks associated with various disaster types (e.g., pandemics, extreme weather events, infrastructure failures) in diverse ways, and these perceptions, in turn, significantly influence their emergency response behaviors. Consequently, risk perception is not solely an intellectual assessment; rather, it’s a highly subjective and context-dependent phenomenon, shaped by individual experiences as well as broader societal and cultural factors.

The relationship between education level and risk perception has garnered significant academic attention. A considerable body of research has indicated that individuals with higher levels of education typically possess a more comprehensive understanding of complex risks and are more inclined to adopt precautionary measures. Bodas et al. [3] demonstrated that individuals with higher educational attainment tend to exhibit greater awareness of disaster risks and show a heightened willingness to engage in preparatory actions for potential hazards. This phenomenon has been observed across diverse cultural contexts, with educated individuals being more proficient at processing risk-related information and taking proactive steps toward disaster preparedness.

However, the influence of education on risk perception is not always straightforward. In certain instances, individuals with lower levels of education may underestimate the risks associated with disasters due to limited access to or understanding of critical information. For example, Ge et al. [4] conducted a study on flood risk perception in Nanjing, China, and found that individuals with higher educational attainment demonstrated a more accurate understanding of flood risks and were more likely to adopt protective measures. In contrast, those with lower levels of education tended to downplay the severity of the threat, resulting in less engagement in preventive behaviors. This underscores the notion that while education can enhance awareness and understanding of risks, it may also contribute to disparities in how different demographic groups perceive and respond to hazards.

Risk perception plays a critical role in shaping individuals’ disaster preparedness behaviors. The Theory of Planned Behavior (TPB) has been extensively utilized to examine how risk perception influences preparedness intentions and actions. For instance, Ng [5] employed an extended TPB model to investigate disaster preparedness in a typhoon-prone district of Hong Kong, demonstrating that risk perception had a significant impact on individuals’ preparedness intentions. This relationship was further mediated by subjective norms and perceived behavioral control. Individuals with stronger perceptions of risk were more likely to engage in proactive measures to prepare for potential disasters, especially when they felt greater control over their ability to mitigate the associated risks. Similarly, Fang et al. [6] investigated the relationship between risk perception and resistance behaviors among residents living near chemical industry parks in China. Their findings revealed that individuals’ perceived risks played a pivotal role in determining their willingness to engage in protest or resistance activities. Notably, higher levels of social trust and public engagement were associated with lower perceived risks, which subsequently reduced the likelihood of active resistance. This highlights the importance of not only comprehending risk perception but also considering how social and community factors shape behavioral responses to perceived risks.

In recent years, machine learning techniques have become increasingly prevalent in the analysis of risk perception and disaster response behaviors. These methods provide novel opportunities to uncover complex patterns and relationships within large datasets, offering insights that traditional statistical approaches may fail to detect. ML algorithms, including decision trees and support vector machines (SVM), have been employed to predict individuals’ behaviors in response to disaster risks and to identify key factors such as education, age, and socioeconomic status that influence risk perception and preparedness actions. For instance, in the domain of pandemic risk perception, ML methods have been applied to analyze public attitudes and behaviors in relation to COVID-19. Vieira et al. [7] developed a Pandemic Risk Perception Scale and utilized ML techniques to model various risk dimensions, including infection risk, emotional health risk, and health system risk, as well as their influence on public preparedness behaviors. These approaches facilitate a more nuanced understanding of how individuals from diverse backgrounds (e.g., educational levels, socioeconomic status) perceive and respond to risks. ML can identify contextual variations in risk perception, allowing for the customization of disaster management strategies to specific groups. By integrating demographic data, past experiences, and risk communication, ML models can provide real-time predictions of public behavior, thereby enhancing the efficacy of risk communication and emergency preparedness efforts. Despite significant progress in understanding the relationship between education level, risk perception, and behavioral responses, several gaps persist. Most research has concentrated on specific disaster types, such as natural hazards or pandemics, with limited cross-disaster comparisons. Future studies should explore risk perception across diverse hazard types to identify both commonalities and distinct factors influencing risk awareness and behavior.

While ML holds promise for analyzing large-scale data, its application in risk perception research remains nascent. Further investigation is needed to integrate ML with theoretical frameworks, such as the TPB, to develop comprehensive models that address the complex interactions between education, risk perception, and disaster preparedness. Additionally, more context-specific studies are required to examine how cultural and social factors, alongside education, influence risk perception and response behaviors.

This study aims to address these gaps by examining the impact of education level on risk perception and behavioral response using ML techniques. By constructing predictive models based on 27 factors, we identify key predictors of behavioral intention and preparatory behavior, with a particular emphasis on education level. Additionally, SHAP values are employed to interpret model outputs and reveal the mechanisms through which education influences these behaviors. Our findings contribute to the growing literature on the application of ML in risk research and provide a scientific foundation for the development of targeted risk management policies.

By integrating recent advancements in ML and risk research, this study provides a novel perspective on the role of education in shaping risk-related behaviors and highlights the importance of context-specific evidence in guiding policy and practice.

## 2. Methodology

ML techniques are widely used in environmental risk assessments [8,9], such as flood susceptibility mapping [10], and in community and behavioral risk evaluations, including identifying at-risk students [11]. These applications highlight ML’s potential to reveal complex relationships and improve decision-making in risk-related contexts.

Random Forest and XGBoost are ML algorithms used for regression and classification, both based on ensemble learning to improve performance by combining multiple models. Random Forest, an ensemble method built on decision trees, constructs numerous trees trained on random subsets of data [12]. Each tree makes independent predictions, which are aggregated through voting or averaging to yield the final prediction. This approach enhances robustness and accuracy while reducing overfitting. XGBoost (Extreme Gradient Boosting), based on gradient-boosted trees [13], iteratively builds decision trees that correct the residual errors of the previous tree, optimizing model performance by minimizing a loss function. Known for its high accuracy, efficiency, and ability to handle large datasets, XGBoost excels in predictive tasks.

This allows us to rank feature importance and uncover key influencing factors. By visualizing feature SHAP values and their relationships with feature values, along with interaction plots, we can construct models that highlight the underlying trends.

As shown in Table 1, behavioral intentions and preparatory behaviors serve as dependent variables, while 27 influencing factors are treated as independent variables to build ML predictive models [4,7,14–22], including Random Forest Regression and XGBoost Regression (XGBRegressor). Model performance is assessed using of MAE (Mean Absolute Error), MSE (Mean Squared Error), and R², with the goal of identifying the optimal input for an interpretable model. SHAP (Shapley Additive Explanations) is employed to interpret model predictions, providing insight into how the model arrives at its outcomes [23]. Based on game theory, SHAP calculates the Shapley value for each feature, evaluating its contribution across various feature combinations. This allows us to rank feature importance and uncover key influencing factors. By visualizing feature SHAP values and their relationships with feature values, along with interaction plots, we can construct models that highlight the underlying trends.

**Table 1:** Definition of Machine Learning Variables.

Variable Type	Variable Name	Variable Symbol	Variable Description
Dependent Variable 1	Behavioral Intention	mtotscore	Factor Scores for Behavioral Intention Items
Dependent Variable 2	Preparatory Behavior	ytotscore	Factor Scores for Preparatory Behavior Items
Influencing Factors	Risk Perception	xtotscore	Factor Scores for Risk Perception Items
	Age [14]	Q11	What is your age? A. 18 or younger B. 19-35 C. 36-45 D. 46 ~ 60 E. 61 or older
	Education Level [15]	Q12	What is your highest level of education? A. Junior high school or below B. High school or vocational school C. College or undergraduate D. Master’s degree or higher
	Household Type [16]	Q13	What is your household type? A. Urban B. Rural
	Marital Status [15]	Q14	What is your marital status? A. Single B. Married C. Divorced D. Widowed
	Health Status [4]	Q16	How do you assess your health status? A. Very poor B. Poor C. Average D. Good E. Very good
	Occupation [17]	Q17	What is your employment status? A. Pubic sector (e.g., teacher, doctor, police officer, judge, civil servant) B. Private sector (e.g., self-employed, private business owner, manual laborer) C. Unemployed
	Earthquake Experience [18]	Q19	Have you ever experienced an earthquake? A. Yes B. No
	Housing Type [19]	Q22	What is your housing type? A. Rented B. Owned C. Self-built D. Other (e.g., irregular land usage)
	Satisfaction with the Timeliness of Local Treatment [7]	Q23	How satisfied are you with the timeliness of treatment near your residence? A. Very dissatisfied B. Dissatisfied C. Neutral D. Satisfied E. Very satisfied
	Province of Residence [20]	Q24	Where is your residence located? [Open-ended]
	Information Access via Authoritative Media [15]	Q25-1	How do you primarily obtain information about earthquake-related issues in your area? [Select all that apply] A. Television, news, and other authoritative media B. Social media (e.g., Weibo, WeChat) C. Propaganda from work or community organizations D. Other
	Information Access via Social Media [15]	Q25-2	
	Information Access via Community Organizations [15]	Q25-3	
	Information Access Via Other Channels [15]	Q25-4	
	Credibility of Official (National/Local) Information [21]	Q26	How credible do you find official (national/local) information sources? A. Very unreliable B. Unreliable C. Neutral D. Reliable E. Very reliable
	Transparency of Earthquake Information [21]	Q28-1	How would you rate the transparency of earthquake information in your area (e.g., causes, aftershock probabilities, impact range)? A. Very dissatisfied B. Dissatisfied C. Neutral D. Satisfied E. Very satisfied
	Transparency of Casualty and Economic Loss Data [21]	Q28-2	How would you rate the transparency of earthquake casualty and economic loss information in your area? A. Very dissatisfied B. Dissatisfied C. Neutral D. Satisfied E. Very satisfied
	Transparency of Earthquake Relief Progress [21]	Q28-3	How would you rate the transparency of earthquake relief progress in your area? A. Very dissatisfied B. Dissatisfied C. Neutral D. Satisfied E. Very satisfied
	Transparency of Earthquake Research Progress [21]	Q28-4	How would you rate the transparency of earthquake disaster relief research progress in your area? A. Very dissatisfied B. Dissatisfied C. Neutral D. Satisfied E. Very satisfied
	Trust in Friends During Earthquakes [22]	Q29-2	During an earthquake, how much do you trust the following individuals or institutions? - Friends: A. Very untrustworthy B. Untrustworthy C. Neutral D. Trustworthy E. Very trustworthy
	Trust in Neighbors During Earthquakes [22]	Q29-3	During an earthquake, how much do you trust the following individuals or institutions? - Neighbors: A. Very untrustworthy B. Untrustworthy C. Neutral D. Trustworthy E. Very trustworthy
	Trust in Colleagues During Earthquakes [22]	Q29-4	During an earthquake, how much do you trust the following individuals or institutions? - Colleagues: A. Very untrustworthy B. Untrustworthy C. Neutral D. Trustworthy E. Very trustworthy
	Trust in Volunteers During Earthquakes [22]	Q29-5	During an earthquake, how much do you trust the following individuals or institutions? - Volunteers: A. Very untrustworthy B. Untrustworthy C. Neutral D. Trustworthy E. Very trustworthy
	Trust in Media Organizations During Earthquakes [22]	Q29-6	During an earthquake, how much do you trust the following individuals or institutions? - Media organizations: A. Very untrustworthy B. Untrustworthy C. Neutral D. Trustworthy E. Very trustworthy
	Trust in Government Agencies During Earthquakes [22]	Q29-7	During an earthquake, how much do you trust the following individuals or institutions? - Government agencies: A. Very untrustworthy B. Untrustworthy C. Neutral D. Trustworthy E. Very trustworthy
	Trust in NGOs During Earthquakes [22]	Q29-8	During an earthquake, how much do you trust the following individuals or institutions? - Non-Governmental organizations (NGOs): A. Very untrustworthy B. Untrustworthy C. Neutral D. Trustworthy E. Very trustworthy

The data for this study were collected from a questionnaire survey conducted on Wenjuanxing between March 7 and March 9, 2024, yielding 4,507 responses. To ensure data quality, samples with completion times under 200 seconds or over 420 seconds, as well as cases with missing variables, were excluded, resulting in a final dataset of 2,239 valid samples.

## 3. Results

### 3.1 Model training and evaluation

The dataset is split into training and validation sets in an 8:2 ratio. Model performance is evaluated using of MAE, MSE, and R^2^, as shown in Table 2. MAE and MSE measure the error between observed and predicted values, with smaller values indicating lower error. R^2^ reflects the goodness of fit, with higher values signifying better model performance. Based on these metrics, the random forest model demonstrates superior performance and will be used in subsequent experiments.

**Table 2:** Evaluation of Machine Learning Performance.

		MAE	MSE	R^2^
ytotscore	RF	0.4861	0.3506	0.2571
	XGB	0.5090	0.3951	0.1629
mtotscore	RF	0.5287	0.4577	0.1419
	XGB	0.5597	0.5277	0.0106

Although the selected variables explain only 25.71% of the variance in the preparatory behavior model (ytotscore) and less than 20% in the behavioral intention model (mtotscore), falling short of fully accounting for the output variables in the random forest model, the primary aim of this section is to identify specific scenarios by examining internal influencing factors within a general context, rather than predicting the output variables. Thus, the goodness of fit serves only as a criterion for model selection and does not impede the exploration of specific scenarios in subsequent analyses.

### 3.2 Results description

**Fig 1:**
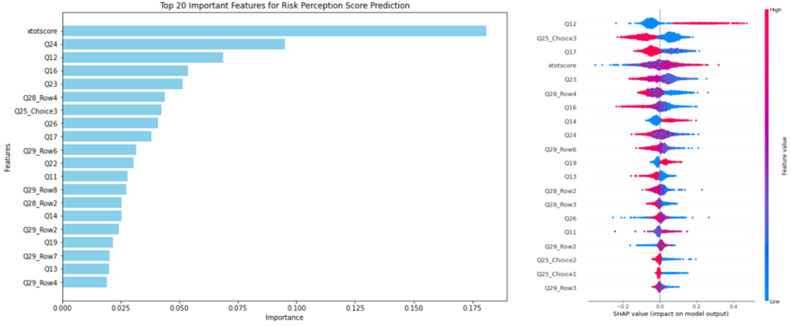
Feature Impact Summary Based on SHAP Values. left: (a) Feature Importance Ranking; Right: (b) Influence of Features on Preparatory Behavior Effectiveness.

As shown in Fig 1, with preparatory behavior as the output variable, Fig 1(a) presents the global feature importance ranking based on the average absolute Shapley values. It’s clear that among the top 20 ranked features, risk perception (xtotscore), place of residence (Q24), and education level (Q12) are particularly significant. To explore how these features influence preparatory behavior, Fig 1(b) displays a scatter plot of SHAP values for each sample and feature. Each point in the plot conveys three pieces of information: the vertical axis represents the feature, the color indicates the feature value (with red indicating higher values and blue values), and the horizontal axis shows the direction of influence on the predicted value.From the feature importance ranking and the influence of each feature on preparatory behavior, we draw the following conclusion.

Education level (Q12) plays a significant role in shaping preparatory behavior (ytotscore), serving as both a core predictive indicator and a potential contextual factor. Firstly, within the multivariate explanatory variables for preparatory behavior, education level ranks third, contributing 6.87% to the overall model, highlighting its strong explanatory power. Secondly, unlike other variables, which show concentrated SHAP values around zero, education level exhibits distinct dispersion. Specifically, individuals with higher educational attainment (red points) show a strong positive correlation with preparatory behavior, while those with lower educational attainment (blue points) display a negative correlation. This further emphasizes the importance of education level in the model and warrants further exploration of its influencing mechanisms [24]. Additionally, as noted by Woodridge, the interaction of significant variables during evidence generation can notably affect the effect size, suggesting that the role of education level as a control variable can directly impact the outcome, making it a critical factor in shaping general evidence.

**Fig 2:**
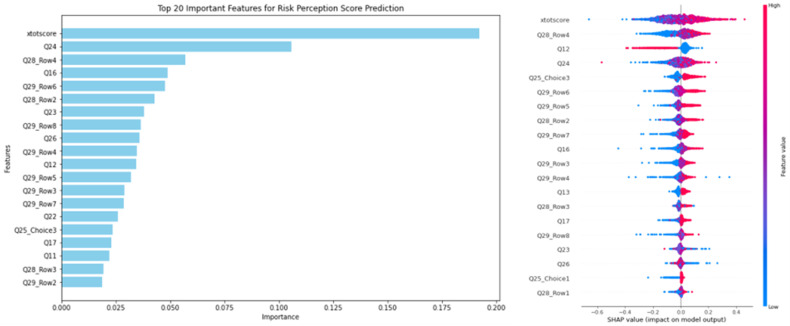
Feature Impact Summary Based on SHAP Values. Left: (a) Feature Importance Ranking; Right: (b) Influence of Features on Behavioral Intention Effectiveness.

As shown in Fig 2, with behavioral intention as the output variable, Fig 2(a) presents the global feature importance ranking based on the average absolute Shapley values for each feature. Among the top 20 features, risk perception (xtotscore), place of residence (Q24), and transparency of research progress on earthquake relief and disaster prevention in the residential area (Q28_Row4) are notably significant. To explore how these features influence preparatory behavior, Fig 2(b) displays a scatter plot of SHAP values for each sample and feature. Each point represents three elements: the vertical axis indicates the feature, the color reflects the feature value (with red representing higher values and blue lower values), and the horizontal axis shows the direction of influence on the predicted value. Based on the feature importance ranking and the impact of each feature on preparatory behavior, the following conclusion can be drawn.

The education level plays a crucial role in predicting behavioral intention and serves as a significant contextual factor. Although its explanatory power is somewhat reduced in the behavioral intention model, it remains moderate. Unlike many variables, which cluster around a SHAP value of zero, the distribution of education level shows distinct patterns.

### 3.3 Examination of contextual evidence

Based on ML model results, education level (Q12) emerges as a significant contextual factor. To validate this, a statistical test is conducted to assess its impact on preparatory behavior. The effect size is analyzed in two groups: one with education level as a control variable and one without. In the group where education level is included, the average effect size is 0.0733; in the group without it, the average effect size is 0.0764. Given education’s potential importance in specific contexts, we hypothesize a significant difference between these groups, which is tested using a t-test.

Before performing the t-test, it is essential to test for variance homogeneity, as this is a prerequisite. The homogeneity test shows a significant difference in variances between the two groups, statistically significant at the 1% level. Therefore, we use the t-test for unequal variances. The results indicate that the mean effect size in the group without education level as a control is significantly higher than in the group with it, with statistical significance at the 1% level. This strongly supports the conclusion that education level plays a crucial role in the relationship between risk perception and preparatory behavior.

To assess the impact of risk perception on behavioral intention, the original evidence is divided into two groups: one that includes education level as a control variable and one that does not. The group considering education level has a mean effect size of 0.1727, while the other group has a mean effect size of 0.1725. If education level is a significant contextual factor, a notable difference should exist between the two groups. A t-test is conducted to examine this. Prior to the t-test, the homogeneity of variances must be verified. The results indicate a significant variances difference at the 1% level, prompting the use of a t-test for unequal variances. The findings show that the mean effect size with educating level as a control is significantly higher than without it.

## 4. Discussion

In summary, education level is a critical contextual factor for several reasons. First, individuals with higher education typically possess enhanced knowledge and information-processing skills [25], enabling them to better understand and assess risks, leading to more effective preparatory measures.

Second, education strengthens risk awareness, with more educated individuals being more vigilant and proactive in preparing for potential risks [26].

Third, higher education fosters logical thinking and critical analysis, allowing individuals to assess situations calmly, develop targeted response strategies, and implement effective preparatory behaviors.

Finally, individuals with higher education are more adept at acquiring and utilizing resources, leveraging information, expert advice, and social networks to manage risks.

## 5. Conclusion

This study contributes to the growing literature on machine learning in risk research, offering actionable insights to improve public preparedness and resilience.

This study examined the impact of education level on risk perception and behavioral response using advanced ML techniques, including Random Forest and XGBoost, with SHAP values for interpretability. The findings highlight education level as a key contextual factor, with higher education linked to more proactive preparatory behaviors and refined risk perceptions. Although the models’ predictive accuracy was limited, they effectively identified key factors and emphasized the role of education in shaping risk-related decisions. These insights underscore the importance of context-specific evidence in risk management and policy formulation. Future research should expand on these findings by incorporating larger datasets and exploring additional contextual variables to further refine our understanding of risk perception. This study contributes to the growing literature on ML in risk research, offering actionable insights to improve public preparedness and resilience.

## Supporting information

S1 File Data. (XLSX)
